# Lipidomic Profiling of *Saccharomyces cerevisiae* and *Zygosaccharomyces bailii* Reveals Critical Changes in Lipid Composition in Response to Acetic Acid Stress

**DOI:** 10.1371/journal.pone.0073936

**Published:** 2013-09-04

**Authors:** Lina Lindberg, Aline XS. Santos, Howard Riezman, Lisbeth Olsson, Maurizio Bettiga

**Affiliations:** 1 Department of Chemical and Biological Engineering, Industrial Biotechnology, Chalmers University of Technology, Gothenburg, Sweden; 2 Department of Biochemistry, University of Geneva, Geneva, Switzerland; 3 Department of Biochemistry, NCCR Chemical Biology, University of Geneva, Geneva, Switzerland; Governmental Technical Research Centre of Finland, Finland

## Abstract

When using microorganisms as cell factories in the production of bio-based fuels or chemicals from lignocellulosic hydrolysate, inhibitory concentrations of acetic acid, released from the biomass, reduce the production rate. The undissociated form of acetic acid enters the cell by passive diffusion across the lipid bilayer, mediating toxic effects inside the cell. In order to elucidate a possible link between lipid composition and acetic acid stress, the present study presents detailed lipidomic profiling of the major lipid species found in the plasma membrane, including glycerophospholipids, sphingolipids and sterols, in *Saccharomyces cerevisiae* (CEN.PK 113_7D) and *Zygosaccharomyces bailii* (CBS7555) cultured with acetic acid. Detailed physiological characterization of the response of the two yeasts to acetic acid has also been performed in aerobic batch cultivations using bioreactors. Physiological characterization revealed, as expected, that *Z. bailii* is more tolerant to acetic acid than *S. cerevisiae. Z. bailii grew* at acetic acid concentrations above 24 g L^−1^, while limited growth of *S. cerevisiae* was observed after 11 h when cultured with only 12 g L^−1^ acetic acid. Detailed lipidomic profiling using electrospray ionization, multiple-reaction-monitoring mass spectrometry (ESI-MRM-MS) showed remarkable changes in the glycerophospholipid composition of *Z. bailii*, including an increase in saturated glycerophospholipids and considerable increases in complex sphingolipids in both *S. cerevisiae* (IPC 6.2×, MIPC 9.1×, M(IP)_2_C 2.2×) and *Z. bailii* (IPC 4.9×, MIPC 2.7×, M(IP)_2_C 2.7×), when cultured with acetic acid. In addition, the basal level of complex sphingolipids was significantly higher in *Z. bailii* than in *S. cerevisiae*, further emphasizing the proposed link between lipid saturation, high sphingolipid levels and acetic acid tolerance. The results also suggest that acetic acid tolerance is associated with the ability of a given strain to generate large rearrangements in its lipid profile.

## Introduction

Biomass-based processes are expected to contribute substantially to the future supply of fuels, chemicals and materials. However, several hurdles must be overcome before an economically feasible lignocellulose conversion bio-process can be achieved, using the yeast *Saccharomyces cerevisiae* to ferment all sugars to valuable products [1]. When lignocellulose is hydrolyzed to release fermentable sugars, inhibitors such as furans and weak organic acids are produced as sugar degradation products, severely affecting the productivity of the microorganism [2]. Acetic acid is formed when the acetyl groups linked to the hemicellulose chains are released; the amount depending on pretreatment type and amount of hemicellulose present in the raw material. Previous studies have reported acetic acid concentrations commonly in the range of 5–10 g L^−1^, although acetic acid concentrations as high as 17.5 g L^−1^ have also been observed [3]. Weak organic acids have been investigated as an alternative to mineral acids for the impregnation of the biomass before pretreatment. This requires further increased tolerance of the fermenting organism, when the sugar streams from hydrolyzed biomass are used as a platform for different fermentation processes [4].

The effect of acetic acid on *S. cerevisiae* has been widely investigated. Reduction in intracellular pH [5], which is detrimental to many cellular processes, including the activity of glycolytic enzymes [6], and acetic-acid-mediated inhibition of NADH dehydrogenase [7] are two of the effects suggested to be responsible for its toxicity. Accumulation of the acetate anion has also been indicated as a possible cause of its toxicity [8], although it was later suggested that the intracellular accumulation of this anion in *S. cerevisiae* may not be as harmful as the anion of some other weak organic acids [5]. Finally, signaling events triggering programmed cell death in response to acetic acid have also been widely described [9].

Regardless of the specific molecular mechanism of the intracellular effect of acetic acid in *S. cerevisiae*, it appears that if this acid could be prevented from entering the cell, its toxic effects could be decreased or eliminated. Acetic acid in its undissociated form has biophysical properties that enable it to passively diffuse into the cell through the lipid bilayer. Passive diffusion has been considered for a long time to be a major contributor to acetic acid uptake by *S. cerevisiae* cells [10], although some recent publications suggest that the major route for acetic acid entry is through the Fps1 aquaglyceroporin channel responsible for glycerol transport [11]. Entry through Fps1 also occurs in a passive diffusive manner, in which only the undissociated form of the acid is able to pass through the channel, due to steric limitations [12]. The similarity in mechanism between the two modes of entry complicates the interpretation of previous studies demonstrating passive diffusion across the lipid bilayer, since both modes have similar pH-dependent uptake kinetics. However, Fps1 is actively degraded in the presence of acetic acid [11] and, consequently, passive diffusion across the lipid bilayer will be an important mode of acetic acid transport, at least during long-term acetic acid exposure. Acetic acid dissociates in the cytosol due to the higher cytosolic pH, and the protons released are pumped out of the cell by the essential ATPase Pma1 [13]. The anion can in turn be extruded by the ABC transporter Pdr12 [14], which function has been shown to be sensitive to the lipid composition of the plasma membrane [15].

Previous strategies to improve acetic acid tolerance include targeted approaches such as deletion of the Fps1 protein to reduce acetic acid uptake [11], and overexpression of the *ELO1* gene involved in the elongation of fatty acids [16] to obtain a membrane with longer fatty acid chains. Non-targeted approaches have also been investigated, such as genome shuffling [17], [18], evolutionary engineering strategies using repeated batch and continuous cultures [19] and evolution in cytostat [20], [21]. All these strategies contribute to increase the acetic acid tolerance, although more remains to be done before acetic acid can be considered harmless to *S. cerevisiae*. New strategies, in combination with those described above, may address the issue better, further increasing the process efficiency when using lignocellulosic substrates containing acetic acid.

Acetic-acid-tolerant species could provide us with valuable information in our effort to understand and overcome acetic acid toxicity. *Zygosaccharomyces bailii*, a common food spoilage yeast [22], is typically isolated from acetic-acid-rich environments such as vinegar or pickles, and may provide a suitable model for studying acetic acid resistance. *Z. bailii* has been extensively investigated from a food science perspective in the development of food preservatives [23] and has also been proposed as a suitable host for the production of organic acids [24], [25]. Acetic acid resistance in *Z. bailii* has previously been explained by a specific acetic acid transporter supporting growth on acetic acid, even in the presence of glucose [26]. Furthermore, higher metabolic flux through ZbAcs2 acetyl-CoA synthetase [27] than in *S. cerevisiae* gives it the ability to consume intracellular acetic acid rapidly. Studies have also demonstrated that *Z. bailii,* unlike *S. cerevisiae,* retains its intracellular pH [28] and plasma membrane integrity [29] upon exposure to acetic acid, which could be due to a difference in membrane lipid composition.

The lipid profile of *S. cerevisiae* has been investigated in great detail [30], [31], but the lipidome response to acetic acid has only been described for one of the three major types of membrane lipids in yeast, namely the glycerophospholipids [16]. Limited data on glycerophospholipid composition are available for *Z. bailii* [32]. Besides the glycerophospholipids, the plasma membrane of *S. cerevisiae* is also composed of approximately 30% sphingolipids [33] and a significant amount of sterols, although the specific amounts are still the subject of discussion [34]. Glycerophospholipids and complex sphingolipids can be further divided, according to their head group, into the different classes listed in Table 1. Different polar head groups, fatty acid chain length and degree of unsaturation give more than 200 different lipid species in *S. cerevisiae* [35].

**Table 1:** Lipid classes in the plasma membrane of *S. cerevisiae*.

Lipid class	Abbreviation
***Glycerophospholipids***	***GPL***
Phosphatidylcholine	PC
Phosphatidylethanolamine	PE
Phosphatidylinositol	PI
Phosphatidylserine	PS
Phosphatidic acid	PA
Phosphatidylglycerol	PG
***Sphingolipids***	***SL***
Ceramide	CER
Inositol phosphate ceramide	IPC
Mannosyl-inositol phosphate-ceramide	MIPC
Mannosyl-di-inositol phosphate-ceramide	M(IP)_2_C
***Sterols***	
Ergosterol	Erg

Main lipid classes (italics) including subclasses.

Cells alter their membrane composition in response to many different stimuli [30]. In order to understand how membranes adapt in relation to stress, detailed information about the lipidome and its flexibility is needed. Recent advances in mass spectrometry have paved the way for more detailed lipidomic studies [36]. However, a general problem associated with investigations of lipid composition in relation to a specific cellular characteristic, is the limitation to analysis of primarily glycerophospholipids and/or sterols, despite the fact that it is widely accepted that sphingolipids are also important components of the plasma membrane [34].

In the present study, we investigated the impact of acetic acid on the physiology and lipidome of *S. cerevisiae* and *Z. bailii*. The physiological response to acetic acid was explored in the two yeasts, in order to create the solid foundation of knowledge required to correctly understand and interpret the lipidomic profiles of the yeasts in the absence and presence of acetic acid. The final goal of the study was to formulate a strategy to improve acetic acid tolerance in *S. cerevisiae*, where the lipid composition is believed to play an important role. The results revealed large lipidomic changes in *Z. bailii* upon acetic acid exposure, while smaller lipidomic changes were observed in *S. cerevisiae.* A higher degree of saturation of the glycerophospholipids and increased amounts of complex sphingolipids were the most striking changes in the adaptation of *Z. bailii* to acetic acid.

## Materials and Methods

### Cell Cultivation

#### Strains and cultivation media

*S. cerevisiae* strain CEN.PK 113_7D (*MATa SUC2 MAL2-8 c*, Scientific Research and Development GmbH, Germany) and *Z. bailii* strain CBS 7555 (Centraalbureau voor Schimmelcultures (CBS) Fungal Biodiversity Centre strain collection, the Netherlands) were used in this study. They were stored at −80°C in YPD glycerol stock (20 g L^−1^ peptone, 10 g L^−1^ yeast extract, 20 g L^−1^ glucose, 400 g L^−1^ glycerol). In preparation for liquid cultivation, cells were grown on YPD plates (20 g L^−1^ peptone, 10 g L^−1^ yeast extract, 20 g L^−1^ glucose, 20 g L^−1^ agar). All liquid cultures were grown in minimal medium (20 g L^−1^ glucose, 0.5 g L^−1^ (NH_4_)_2_SO_4_, 0.05 g L^−1^ MgSO_4_×7H_2_O, 0.3 g L^−1^ KH_2_PO_4_, 1 mL L^−1^ vitamin solution, 1 mL L^−1^ trace element solution). Vitamin solution and trace element solution were prepared as described previously [10]. In growth experiments in Erlenmeyer flasks, the pH was maintained by the buffering capacity of 50 mM potassium hydrogen phthalate. KOH was used to adjust the pH in the medium to 5. Acetic acid was added to the medium when indicated as a concentrated stock solution adjusted to pH 5 with KOH.

#### Inoculum

Pre-culture for the aerobic batch cultures was prepared by transferring one colony to 10 mL minimal medium in a 100 mL Erlenmeyer flask. For experiments in bioreactors, a second larger pre-culture was used, cultured from cells in the first inoculum. In all cultures greater than 10 mL, baffled Erlenmeyer flasks were used with a culture volume occupying a maximum of 10% of the flask volume. Cultures were grown under continuous shaking at 150 rpm at 30°C overnight. Inoculum for experiments in bioreactors was harvested by centrifugation at 3000×g for 3 min at 20°C and resuspended in 10 mL fresh minimal medium, and aseptically added to the reactor.

#### Aerobic batch cultivations

Initial screening of acetic acid tolerance was carried out in 500 mL baffled Erlenmeyer flasks containing 50 mL minimal medium and acetic acid at a range of concentrations (for details see Table 2). A pre-culture that had reached an OD_600_ of 4 was used to inoculate the cultures, resulting in an initial OD_600_ of 0.2. Cultivation continued under continuous shaking at 150 rpm at 30°C, and growth was monitored by regular sampling for OD_600_ measurements.

**Table 2:** Amounts of acetic acid added to the medium during growth screening.

*S. cerevisiae*		*Z. bailii*	
Total (g L^−1^)	Undissociated^1^ (g L^−1^)	Total (g L^−1^)	Undissociated^1^ (g L^−1^)
0	0	0	0
1.8	0.8	3.0	1.3
2.7	1.2	6.0	2.6
3.6	1.6	7.5	3.3
4.5	2.0	9.0	4.0
6.0	2.6	10.5	4.6
7.5	3.3	12.0	5.3
9.0	4.0	13.5	5.9
10.5	4.6	15.0	6.6
12.0	5.3	16.5	7.3
–	–	18.0	7.9
–	–	21.0	9.2
–	–	24.0	10.6
–	–	27.0	11.9
–	–	30.0	13.2
–	–	33.0	14.5
–	–	36.0	15.8

1 Undissociated form of acetic acid at pH 5.

Bioreactor cultivations were performed in 2 L working volume bioreactors (DASGIP AG, Jülich, Germany). *S. cerevisiae* was cultivated in minimal medium containing 9 g L^−1^ acetic acid, and *Z. bailii* in a similar medium containing 24 g L^−1^ acetic acid. Control cultivations for each yeast were performed under identical conditions, except for the absence of acetic acid. A minimum of three cultivations was performed for each condition. Pre-cultures that had reached late exponential phase were used to inoculate the bioreactors, resulting in an initial OD_600_ of 0.2. The temperature was maintained at 30°C, and the pH at 5 by the addition of KOH and HCl. The bioreactors were sparged with a constant flow of air at 0.5 VVM. The minimum dissolved oxygen tension (DOT) was set to 40% of fully air-saturated conditions, and was automatically controlled by changing the stirring speed. The initial stirring speed was 400 rpm. When foaming occurred, drops of Antifoam (Antifoam 204, Sigma Aldrich) were added to the medium. The concentrations of oxygen and carbon dioxide in the air at the inlet and in the off-gas were monitored with on-line gas analyzers (DASGIP AG, Jülich, Germany). Water vapor in the off-gas was compensated for using the Magnus formula [37]. Samples were withdrawn regularly for measurements of OD_600_, dry weight and extracellular metabolites.

#### Dry cell weight

The dry weight of the cell mass was determined in duplicate by filtering 5–20 mL of culture broth through dry, pre-weighed 0.45 µm PES membranes (Sartorius Stedim, Aubagne, France). The cells were washed with deionized water, dried in a microwave oven at 120 W for 15 min, and further dried in a silica-gel desiccator for a minimum of 24 hours before weighing.

#### Analysis of extracellular metabolites

Samples removed from the culture were immediately filtered through 0.2 µm nylon syringe filters (VWR International, West Chester, PA, USA) and stored at −20°C until analysis. Extracellular metabolites (glucose, ethanol, glycerol, acetic acid, succinate and pyruvate) were analyzed with HPLC (Ultimate 3000, Dionex, Sunnyvale, CA, USA). An Aminex HPX87-H column (Bio-Rad Laboratories, Munich, Germany) maintained at 45°C was used for separation with a mobile phase of 5 mM H_2_SO_4_ at a flow rate of 0.6 L min^−1^. Glucose, ethanol and glycerol were detected using a refractive index detector (Shodex RI-101, Showa Denko, New York, NY, USA). Acetic acid, succinate and pyruvate were detected with a UV detector at 210 nm (Ultimate 3000, Dionex, Sunnyvale, CA, USA). Metabolites were quantified using a standard curve with a minimum of 6 points of known concentrations within the expected range of the unknown samples.

#### Calculation of physiological parameters

All data are presented as mean values of at least three biological replicates ± standard deviation.

A standard cell mass composition of CH_1.8_O_0.5_N_0.2_ was assumed in all calculations for both microorganisms used in this study [38]. The maximum specific growth rate, µ_max_, was calculated after identification of the exponential growth phase. The length of the lag phase was calculated based on the equation for exponential growth, and the concept that if a lag phase occurs, the regression of exponential growth will exhibit a theoretical initial biomass value lower than that determined experimentally. This concept, presented in Equation 1, was used to calculate the length of the lag phase:
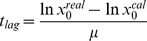
(1)where x_0_^real^ is the measured initial biomass concentration, and x_0_^cal^ is the calculated theoretical initial biomass concentration derived from linear regression of the natural logarithm of biomass versus time.

The yield (Y_i/s_) from the total consumed substrate (glucose+acetic acid) was calculated during the exponential growth phase by plotting the compound of interest i, versus total consumed substrate and calculating the derivative of the linear relation (Equation 2).
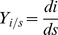
(2)


Biomass-specific substrate consumption and product production rates, q_i_, were calculated during exponential growth, using the yield of the compound of interest i on biomass x as the proportionality factor between q_i_, and the maximum specific growth rate ( = biomass-specific biomass production rate), according to Equation 3.
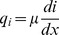
(3)


All data were corrected for the evaporation of ethanol. Corrections were made assuming that the rate of evaporation was 1% of the ethanol present at each point in time.

### Lipidomic Profiling

#### Cell samples for lipid analysis

Quadruplicate aerobic batch bioreactor cultivations were performed using the two yeasts in the presence and absence of acetic acid, using a setup identical to that used for the physiological study (see “Aerobic batch cultivations”). A volume of 250 mL culture was harvested during the mid-exponential growth phase (when the cultures had a residual glucose concentration in the range 7.2–10.3 g L^−1^), and centrifuged at 3000×g for 4 min at 4°C. The cell pellet was resuspended in 40 mL of the supernatant, aliquoted to four 15 mL Falcon tubes, and centrifuged again. The supernatant was discarded and the cell pellet was frozen in liquid nitrogen before storage at −80°C.

#### Lipid extraction

- Glycerophospholipids and sphingolipids. Lipids were extracted as described previously [39] with minor modifications. Briefly, 12.5 OD of cells (OD600 units) were re-suspended in 3 mL of extraction solvent [ethanol, water, diethyl ether, pyridine, and 4.2N ammonium hydroxide (15∶15:5∶1:0.18, v/v)]. Internal standards were added (PC31∶1 3.75 nmol, PE31∶1 3.75 nmol, PI31∶1 3.0 nmol, PS31∶1 2.0 nmol, C17Cer 0.6 nmol and C8GC 1 nmol) and the extraction was performed as described previously [39].
- Sterols. The cells (12.5 OD600 units) were resuspended in 500 µL water and 750 µL methanol. Cholesterol (10 nmol) was used as internal standard. Cells were vortexed vigorously for 1 minute, after which 1500 µL chloroform was added. Vigorous vortexing for 6 minutes and centrifuged at 4000 rpm for 10 minutes. The upper aqueous phase was discarded and the organic phase was collected. Samples were then cleaned using an SPE column (Chromabond SiOH 0.1 g, Macherey-Nagel, Düren, Germany) [39], dried and flushed with nitrogen before storage to avoid oxidation [39].

#### Lipid analysis

- Glycerophospholipids and sphingolipids. Lipid analysis was performed by multiple reaction monitoring with a TSQ Vantage Triple Stage Quadrupole Mass Spectrometer (Thermo Scientific, Whaltman, MA, USA) equipped with a robotic nanoflow ion source, Nanomate (Advion Biosciences, Ithaca, NY, USA). Lipids were quantified relative to the appropriate internal standard, as described previously [39], and normalized to the total amount of phosphate in each sample. Four independent biological replicates were analyzed, each of which comprised 3 to 6 technical replicates. Results are expressed as apparent quantities in arbitrary units (a.u.). The arbitrary units of glycerophospholipids are comparable within the different classes, but are not comparable with the arbitrary units of sphingolipids. As no standards for the three classes of sphingolipids were available, their absolute quantities could not be determined.
- Sterols. Extracts were analyzed with GC-MS, as described previously [39].

The lipid analysis raw data from all four biological replicates including mean, median, standard deviation and variance values of the replicates are available in Data S1.

## Results

The response of *S. cerevisiae* and *Z. bailii* to acetic acid was investigated at a strain-specific acetic acid concentration that exposed them to a comparable level of stress, i.e. a level of acetic acid that resulted in half the specific growth rate compared with the case when no acetic acid was added. Four conditions were investigated: *S. cerevisiae* and *Z. bailii* under control conditions, and under the strain-specific stress concentration of acetic acid. Therefore, a double comparison will be presented: *Z. bailii* in relation to *S. cerevisiae*, and cells stressed with acetic acid in comparison to the control condition.

### Growth Screening of Acetic Acid Tolerance in *S. cerevisiae* and *Z. bailii*

To map the effect of increasing concentrations of acetic acid in *S. cerevisiae* and *Z. bailii*, an initial growth screening was performed to identify for each of the two yeasts, the acetic acid concentration that resulted in comparable levels of stress to the cells. The level of stress was defined as a 50% reduction in the maximum specific growth rate (µ_max_) compared to the maximum specific growth rate achieved in cultures without the addition of acetic acid. Cells were grown under aerobic conditions in shake flasks using minimal medium at pH 5 containing 0–12 g L^−1^ acetic acid for *S. cerevisiae* and 0–36 g L^−1^ acetic acid for *Z. bailii*. The acetic acid concentrations, including the amount of undissociated acid at pH 5, are presented in Table 2. In *S. cerevisiae*, 9 g L^−1^ acetic acid caused a reduction in µ_max_ of 45%, while at 12 g L^−1^ very limited growth was observed after 11 hours. In *Z. bailii*, 12 g L^−1^ acetic acid only reduced µ_max_ by 28%, and 24 g L^−1^ led to a 41% reduction in µ_max_. Therefore, for further work, it was decided that *S. cerevisiae* was cultured in a medium containing 9 g L^−1^ acetic acid, and *Z. bailii* in a medium containing 24 g L^−1^, to expose the cells to a moderate and comparable level of stress. At pH 5, this corresponds to 4.0 g L^−1^ and 10.6 g L^−1^ of the inhibitory undissociated form of the acid for *S. cerevisiae* and *Z. bailii,* respectively.

During this experiment, phthalate buffer was used to control the pH. However, due to the ability of *Z. bailii* to consume all the supplied acetic acid, the buffering capacity was not sufficient, and the pH increased to 8.9. This increase in pH initially relieved the acetic acid stress, by decreasing the amount of undissociated acetic acid, although above certain pH values, alkalinity affects the specific growth rate negatively.

### Physiological Response of *S. cerevisiae* to 9 g L^−1^ Acetic Acid

Acetic acid stress in *S. cerevisiae* was investigated in aerobic batch cultivations in bioreactors with 9 g L^−1^ acetic acid. Bioreactor cultivation allowed well controlled growth conditions, facilitating the determination of yields and rates. In addition, pH and dissolved oxygen could be kept constant irrespective of the growth phase.

**Figure 1:**
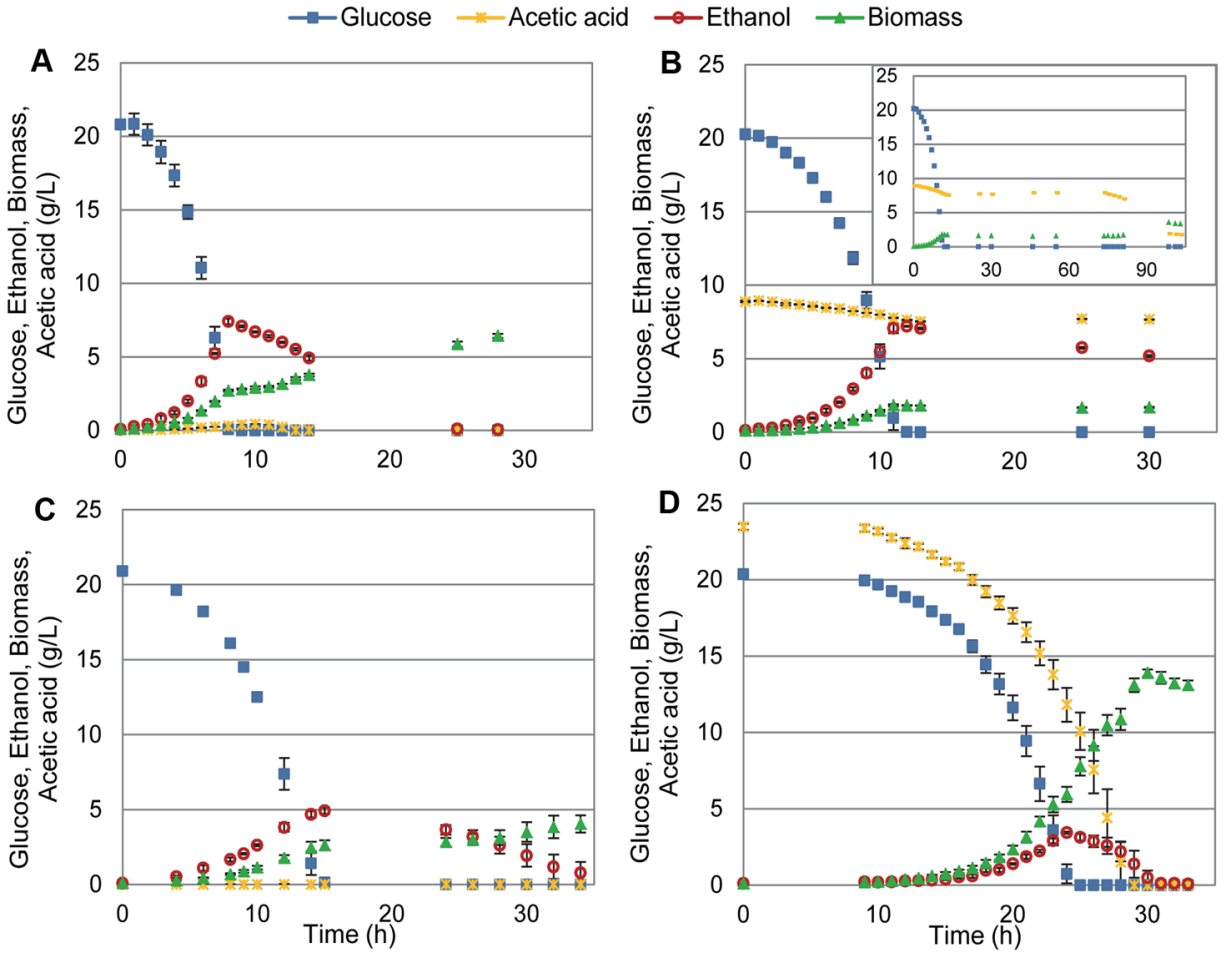
Fermentation profiles of *S. cerevisiae* and *Z. bailii* cultured with and without acetic acid. The graphs show data until all the carbon sources had been utilized. **A**. *S. cerevisiae* cultured in minimal medium. **B.** *S. cerevisiae* cultured in minimal medium with 9 g L^−1^ acetic acid. **C.** *Z. bailii* cultured in minimal medium. **D.** *Z. bailii* cultured in minimal medium with 24 g L^−1^ acetic acid. The graphs represent the mean of n≥3 biological replicates with error bars indicating standard deviation. For the sake of clarity, error bars are omitted from the insert in figure 1B.

*S. cerevisiae* exposed to acetic acid was compared with *S. cerevisiae* cultured without acetic acid (Figure 1A, B). In this experiment, the acetic acid stress resulted in a 33% decrease in µ_max_, and an 8% decrease in the specific glucose consumption rate (q_Glucose_) under the applied conditions (Table 3). The decrease in µ_max_ was smaller than in the screening tests in the Erlenmeyer flasks, probably due to an overall higher specific growth rate in the bioreactors; a common feature of cultures with controlled oxygenation and pH. The biomass yield from the total substrate, Y_x/s_, was slightly lower in acetic acid exposed cultures (Table 3), possibly due to the higher amount of ATP required for extruding protons out of the cell. Another sign of stress was the prolonged lag phase during the diauxic shift after glucose exhaustion, which increased from 2 hours in control cultures to 80 hours in cultures containing acetic acid (Figure 1B). The ethanol yield from total substrate, Y_EtOH/s_, was the same in both examined conditions (Table 3). Acetic acid stress led to a 69% reduction in glycerol yield, while pyruvate and succinate levels were not significantly affected by the addition of acetic acid (Table 3).

**Table 3:** Physiological data obtained from aerobic batch fermentations.

		*S. cerevisiae*		*Z. bailii*	
		Control	9 g L^−1^ acetic acid	Control	24 g L^−1^ acetic acid
***Specific production/consumption rate***					
µ_max, Glucose phase_	h^−1^	0.44±0.01	0.30±0.00	0.24±0.01	0.23±0.01
µ_max, Acetic acid phase_	h^−1^	N/A	N/A	N/A	0.12±0.01
q_Glucose_	cmol×cmol DW^−1^×h^−1^	−2.87±0.02	−2.63±0.06	−1.51±0.14	−0.62±0.02
q_Acetic acid_	cmol×cmol DW^−1^×h^−1^	0.05±0.00	−0.14±0.02	0.00±0.00	−0.37±0.01
***Yield from total substrate***					
Y_x/s_	cmol×cmol^−1^	0.15±0.00	0.11±0.00	0.16±0.02	0.23±0.00
Y_EtOH/s_	cmol×cmol^−1^	0.45±0.01	0.46±0.00	0.38±0.01	0.14±0.01
Y_Acetic acid/s_	cmol×cmol^−1^	0.02±0.00	N/A	0.00±0.00	N/A
Y_Glycerol/s_	cmol×cmol^−1^	0.03±0.00	0.01±0.00	0.05±0.01	0.04±0.04
Y_Pyruvate/s_	cmol×cmol^−1^	0.004±0.000	0.005±0.000	0.005±0.001	0.004±0.000
Y_Succinate/s_	cmol×cmol^−1^	0.000±0.001	0.000±0.000	0.003±0.001	0.000±0.000
***Lag phase before initial growth***					
Lag phase	h	0.25±0.05	1.07±0.06	0.38±0.07	6.77±0.41

*S. cerevisiae* and *Z. bailii* were cultured in minimal medium using bioreactors. Different amounts of acetic acid were added to the medium to stress the microorganisms equally. The results were calculated from at least three biological replicates, and are given as the means ± standard deviation.

Unexpectedly, a slight co-consumption of acetic acid and glucose was observed in *S. cerevisiae* cultures stressed with acetic acid (Figure 1B, Table 3), leading to a decrease in acetic acid concentration from 9 g L^−1^ to 7 g L^−1^ during the 12 hours of glucose consumption. Co-consumption of glucose and acetic acid is not often observed in *S. cerevisiae*, but it has been described for some commercial *S. cerevisiae* wine strains [40], and it is a well-known phenomenon in glucose-limited chemostats [41]. However, acetic acid consumption ceased when the glucose had been exhausted, and resumed only after a lag phase of 80 hours, when it was consumed together with the byproducts, ethanol and glycerol. During the long lag phase, most of the ethanol produced during the glucose phase evaporated, and acetic acid consumption started when only 2 g L^−1^ ethanol was left in the culture (Figure 1B). One could speculate that, since both acetic acid and ethanol are toxic to the cells [42], they might have acted synergistically, preventing growth until the point when at least part of the ethanol had evaporated, which may explain the long lag phase.

### Physiological Response of *Z. bailii* to 24 g L^−1^ Acetic Acid

Acetic acid stress in *Z. bailii* was investigated in aerobic batch cultures in bioreactors with 24 g L^−1^ acetic acid. Cultivations exposed to acetic acid were compared with control cultivations without acetic acid. Cultivation in the presence of acetic acid resulted mainly in a prolonged lag phase, from 0 hours to approximately 7 hours, but no decrease in µ_max_ was observed (Figure 1C, D, Table 3). In the initial screening, a 41% decrease in µ_max_ was observed upon exposure to acetic acid at this concentration. However, the pH was not controlled in the initial screening experiments, which could possibly explain the reduced specific growth rate. Despite the similar values of µ_max_ in the absence and presence of acetic acid, the prolonged lag phase is a strong indication of cellular stress. Once the cells cultured with acetic acid started to grow, co-consumption of glucose and acetic acid occurred, as described elsewhere [43]. In the cultures containing acetic acid, the specific glucose consumption rate, q_Glucose_, was 68% faster than the specific acetic acid consumption rate, q_Acetic acid_, when comparing rates in cmol (Table 3). The overall specific substrate consumption rate (q_Glucose_+q_Acetic acid_) of the stressed cells was 36% lower than q_Glucose_ for cells cultured without acetic acid. Although µ_max_ was not affected by the acetic acid stress (Table 3), q_EtOH_ decreased by 76% (from 0.57±0.06 cmol×cmol DW^−1^×h^−1^ to 0.15±0.01 cmol×cmol DW^−1^×h^−1^), implying a lower overall productivity of the stressed cells. Ethanol yield from the total substrate, Y_EtOH/s_, was 63% lower for the stressed cells (Table 3), and 39% lower when comparing the ethanol yield from consumed glucose instead of the total substrate (glucose+acetic acid) (data not shown). In this case, it may be more relevant to compare the ethanol yield from glucose, since acetic acid consumption is not expected to contribute to ethanol production. The higher Y_x/s_, in the presence of acetic acid is explained by the use of acetic acid as an additional carbon source, as well as the low conversion of substrate to ethanol (Table 3). Less succinate was produced by the stressed cells, while glycerol and pyruvate levels were not significantly affected by the addition of acetic acid.

### Physiological differences between *S. cerevisiae* and *Z. bailii* Exposed to Acetic Acid

As previously concluded [42], *S. cerevisiae* exhibits faster growth than *Z. bailii* when the cells are cultured without acetic acid; µ_max_ was 86% higher and q_Glucose_ was 90% higher using *S. cerevisiae* compared with *Z. bailii* (Table 3). *Z. bailii* exhibited a 16% lower Y_EtOH/s_ than *S. cerevisiae*, probably due to the less extensive overflow metabolism, as described elsewhere [44]. When the cells were exposed to acetic acid, *Z. bailii* exhibited a 69% lower Y_EtOH/s_ than *S. cerevisiae* (Table 3). No significant differences were observed in byproduct profile (acetic acid, glycerol, pyruvate, succinate) between the two yeasts with or without acetic acid.

### Lipidome Adaptation in *S. cerevisiae* in Response to 9 g L^−1^ Acetic Acid

**Figure 2:**
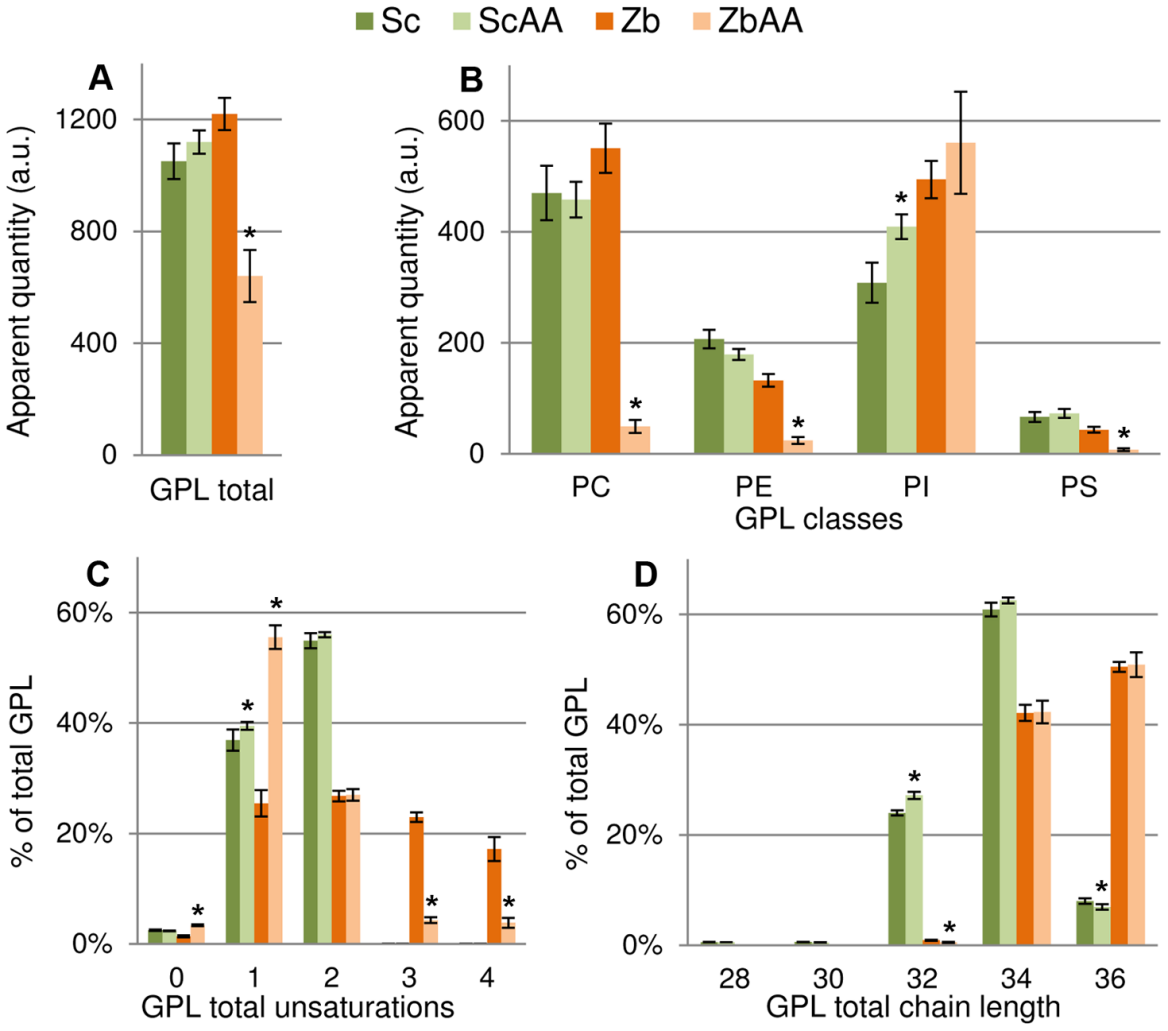
Glycerophospholipid profiles of *S. cerevisiae* and *Z. bailii* in response to acetic acid. Sc: *S. cerevisiae* cultured in minimal medium. ScAA: *S. cerevisiae* cultured in minimal medium with 9 g L^−1^ acetic acid. Zb: *Z. bailii* cultured in minimal medium. ZbAA: *Z. bailii* cultured in minimal medium with 24 g L^−1^ acetic acid. Apparent quantities were calculated relative to the appropriate internal standard, and normalized to the total amount of phosphate in each sample (see Materials and Methods). **A.** Total glycerophospolipids (GPL) analyzed (PC, PE, PI, PS). **B.** Glycerophospholipid classes. **C.** Amount of unsaturations in total glycerophospholipids, presented per lipid, containing two fatty acyl chains. **D.** Total glycerophospholipid chain length, presented per lipid, containing two fatty acyl chains. *Significant difference compared with control condition, obtained by t-tests (P<0.05). The results were calculated from biological replicates (n = 4) and are given as the mean ± standard deviation. For lipid nomenclature, see Table 1.

**Figure 3:**
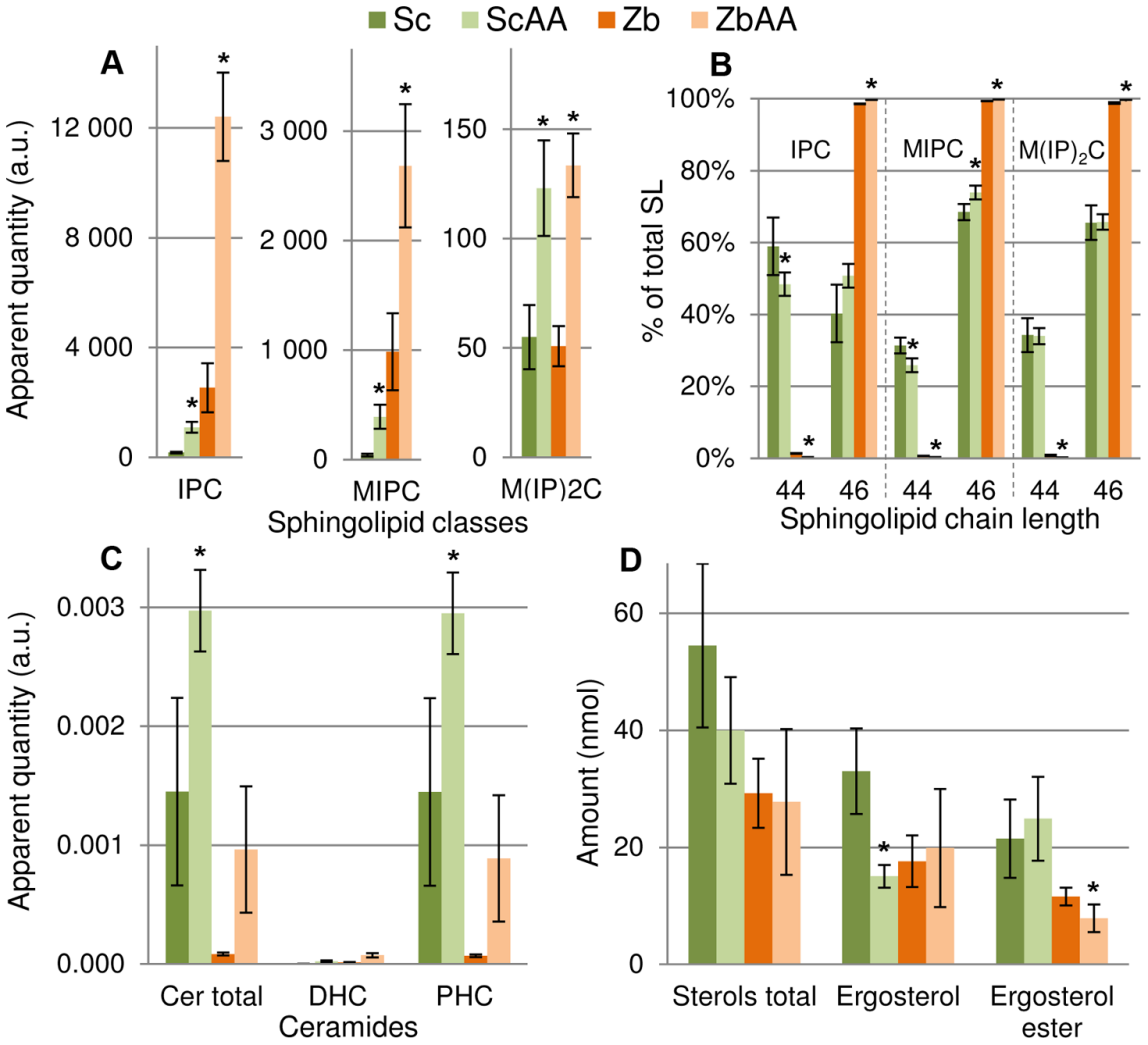
Sphingolipid and sterol profiles of *S. cerevisiae* and *Z. bailii* in response to acetic acid. Sc: *S. cerevisiae* cultured in minimal medium. ScAA: *S. cerevisiae* cultured in minimal medium with 9 g L^−1^ acetic acid. Zb: *Z. bailii* cultured in minimal medium. ZbAA: *Z. bailii* cultured in minimal medium with 24 g L^−1^ acetic acid. Apparent quantities were calculated relative to the appropriate internal standard, and normalized to the total amount of phosphate in each sample (see Materials and Methods). **A.** Head group classes of complex sphingolipids. **B.** Total sphingolipid chain length, presented per lipid, containing two fatty acyl chains. **C.** Total ceramides and ceramide sub-classes. Abbreviations: DHS: dihydrosphingosine, PHS: phytosphingosine. **D.** Ergosterol, ergosterol esters and total sterols. *Significant differences, obtained by t-test (P<0.05) compared with control condition (Sc or Zb). The results were calculated from biological replicates (n = 4) and are given as the mean ± standard deviation. For lipid nomenclature, see Table 1.

**Figure 4:**
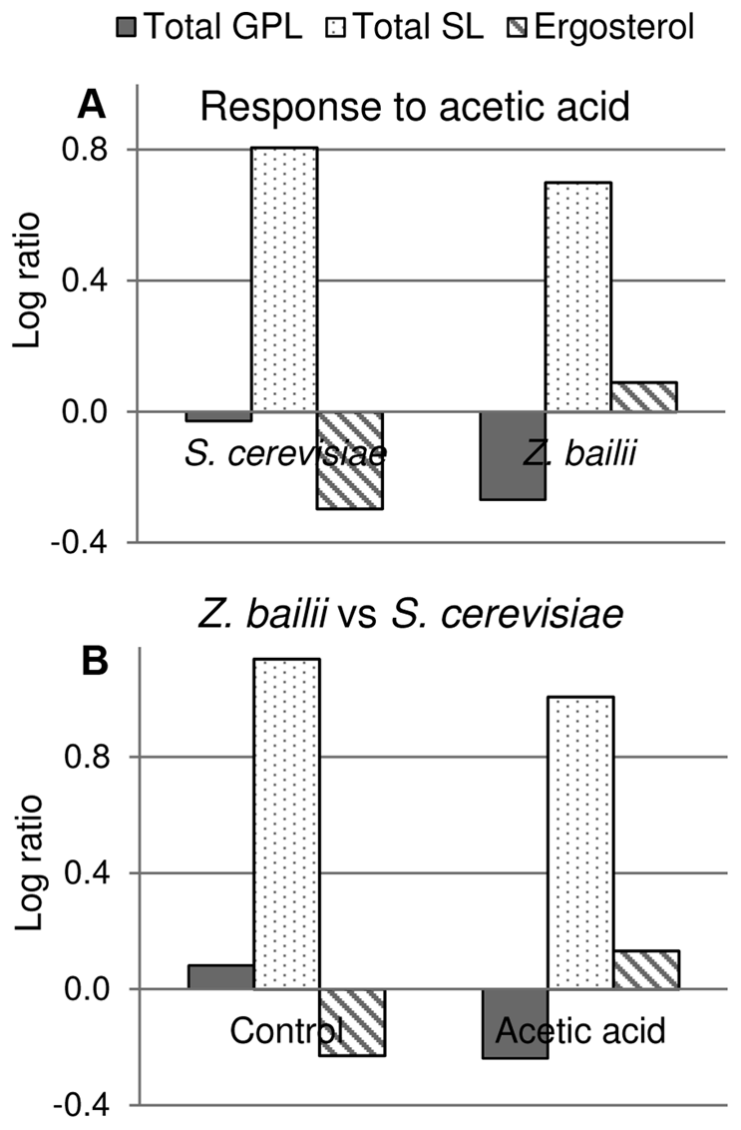
Overall lipidome response of *S. cerevisiae* and *Z bailii* cultured with and without acetic acid. *S. cerevisiae* was cultured with 9 g L^−1^ acetic acid and *Z. bailii* with 24****g L^−1^ acetic acid. **A.** Response of *S. cerevisiae* and *Z. bailii* to acetic acid. **B.** Comparison of *S. cerevisiae* to *Z. bailii* under control conditions and acetic acid stress. Total sphingolipids is an approximation as absolute quantities are not available (see Materials and Methods). The results were calculated from biological replicates (n = 4) and are given as the mean ± standard deviation.

In order to investigate whether *S. cerevisiae* responds to acetic acid by adapting its lipidome, cells were cultivated with 9 g L^−1^ acetic acid and compared with those cultivated without acetic acid stress. The total lipids extracted from cell samples during mid-exponential growth were analyzed with electrospray ionization multiple-reaction-monitoring mass spectrometry (ESI-MRM-MS). When comparing, acetic acid stressed cells, with the control condition, no large significant changes were found regarding glycerophospholipids, when total glycerophospholipids, glycerophospholipid classes, total glycerophospholipid unsaturations or total glycerophospholipid chain length were examined, apart from a slight increase in phosphatidylinositol (PI) in the stressed cells (Figure 2A–D). All glycerophospholipid classes were analyzed in this study except for phosphatidic acid and phosphatidylglycerol, which are usually present at low amounts in the plasma membrane [45]. Remarkably, there was a general increase in the different subgroups of complex sphingolipids: 6.2× IPC, 9.1× MIPC and 2.2× M(IP)_2_C (Figure 3A). Sphingolipid chain length was comparable between the two conditions with almost half of the species containing 44 carbons in the sphingoid base and acyl chain combined, and the rest of the sphingolipids containing 46 carbons in the two chains (Figure 3B). Ceramides, precursors of complex sphingolipids, with low abundance in the plasma membrane, increased by 2.0× in the stressed cells (Figure 3C). Ergosterol, the major sterol present in the plasma membrane, decreased by 0.54×, while a significant change could not be detected for ergosterol esters and total sterols (Figure 3D). The general response of *S. cerevisiae* to acetic acid is illustrated in Figure 4A.

### Lipidome Adaptation in *Z. bailii* in Response to 24 g L^−1^ Acetic Acid

*Z. bailii* cultured with 24 g L^−1^ acetic acid exhibited substantial changes in lipid metabolism compared to cells cultured without acetic acid. Upon exposure to acetic acid, the level of total glycerophospholipids decreased to 0.54× (Figure 2A). Drastic reductions were observed in phosphatidylethanolamine (PE), phosphatidylcholine (PC) and phosphatidylserine (PS), while PI remained high (Figure 2B). The degree of unsaturation of glycerophospholipids was considerably reduced, showing a striking increase in saturated and monounsaturated fatty acyl chains (Figure 2C). Since PI exhibited a lower degree of unsaturation than PE, PC and PS in the control experiment, a significant proportion of the decrease in saturation was due to the decrease in PE, PC, and PS in the cells cultured with acetic acid (Figures S1–S4). No significant difference in glycerophospholipid chain length was observed between stressed and unstressed *Z. bailii* cells (Figure 2D). An increase in total complex sphingolipids was observed, with increases of 4.9×, 2.7× and 2.7× in the specific subgroups IPC, MIPC and M(IP)_2_C, respectively (Figure 3A). *Z. bailii* contained mainly complex sphingolipids with a combined chain length of 46 carbons (Figure 3B). Ceramide levels appeared higher in samples from cells cultured with acetic acid, but the difference was not statistically significant (P<0.05) (Figure 3C). Unlike *S. cerevisiae*, ergosterol levels in *Z. bailii* were unchanged by cultivation with acetic acid, and only a slight decrease in ergosterol esters was observed (Figure 3D). The general response of *Z. bailii* to acetic acid is illustrated in Figure 4A.

### Differences in the Lipidome Profiles of *S. cerevisiae* and *Z. bailii*

The lipidome of *Z. bailii* was found to be very adaptable to acetic acid stress, while less drastic changes were seen in *S. cerevisiae*. When comparing the two yeasts cultured without acetic acid, the amounts of total of glycerophospholipids were equivalent, and the head group profiles were relatively similar, except for a higher PI content and slightly lower PE content in *Z. bailii* (Figure 2A,B). In agreement with the results from a previous study, the glycerophospholipid chain length was, in general, two carbons longer in *Z. bailii,* and the fatty acid chains contained double unsaturations not found in *S. cerevisiae* [32], due to the lack of Δ12 fatty acid desaturase [46] (Figure 2C,D). The most remarkable difference between the two yeasts was the considerable higher basal level of complex sphingolipids in *Z. bailii* compared to *S. cerevisiae* (Figure 3A). IPC was 14.3× higher and MIPC was 22.9× higher, while M(IP)_2_C was 0.9× lower. The average complex sphingolipid chain length was also higher: 46 carbons in *Z. bailii,* while this *S. cerevisiae* strain had almost half with 44 carbons and the rest with 46 carbons (Figure 3B). Total sterol levels in *Z. bailii* were 0.29× lower compared to *S. cerevisiae* (Figure 3D).

Comparison of the response of the two yeasts to acetic acid revealed a slightly smaller increase in complex sphingolipids in *Z. bailii* than in *S. cerevisiae,* although the basal levels are much higher in *Z. bailii* (Figure 3A,4B). Glycerophospholipid levels remained high in *S. cerevisiae* while, in *Z. bailii,* the total amount of glycerophospholipids was 0.54× lower, and the degree of saturation of glycerophospholipids was increased by acetic acid exposure (Figure 2A, 4A). Ergosterol remained unchanged in *Z. bailii*, while it decreased by 0.54× in *S. cerevisiae* upon acetic acid treatment (Figure 3D, 4A). A general comparison of the lipidome in *S. cerevisiae* and *Z. bailii* is presented in Figure 4B. Complete lipid profiles of all lipid species analyzed are presented in Figures S1–S6.

## Discussion

*Z. bailii* has proven to be highly tolerant to acetic acid, in this study as well as in others [42]. Our findings suggest that two physiological characteristics are associated with the higher acetic acid tolerance of *Z. bailii* compared to *S. cerevisiae*. First, the ability of *Z. bailii* to co-consume glucose and acetic acid gave it an intrinsic ability to remove the toxic intracellular acetic acid. This feature is well-known [26], and has been explained by the physiological features of a specific acetate transporter [43] that is not glucose-repressed, in combination with a high activity of ZbAcs2, converting acetic acid to acetyl-CoA inside the cell [27]. *S. cerevisiae* was also able to consume acetic acid together with glucose, but the acetic acid consumption was 2.6× higher in *Z. bailii,* which also continued consuming the remaining acetic acid after the glucose had been depleted. Second, the lower ethanol yield of *Z. bailii* prevents the synergic toxic effect of ethanol and acetic acid. In fact, it has previously been reported that ethanol exacerbates the acetic acid stress to a much lower extent in *Z. bailii* than in *S. cerevisiae* [42] and, therefore, the low ethanol yield of *Z. bailii* is not regarded as a property supporting acetic acid resistance, but rather a further reason why *S. cerevisiae* has poor tolerance to the acid in batch cultivation, equivalent to the conditions used in this study. Nevertheless, the foremost property of *Z. bailii,* which we believe contributes in an important way to its superior acetic acid tolerance, is its ability to undergo major lipidome rearrangements when exposed to acetic acid. This ability, in particular the enrichment of saturated acyl chains arising from glycerophospholipids and complex sphingolipids, may well lead to reduced permeability of the plasma membrane to acetic acid, and saturated acyl chains are known to increase membrane order [47]. Lipidome-wide rearrangements were also seen in *S. cerevisiae*, but they were not as extensive. Based on our observations, we suggest that *Z. bailii* can withstand acetic acid due to changes leading to an adapted plasma membrane with low acetic acid permeability, and that it regulates acetic acid uptake through active transport. Further evidence is provided by a previous study on acetic acid transport in *Z. bailii* [26], in which a dual-term model for transport kinetics was proposed, including a Michaelis-Menten and a first-order kinetics term, the former suggesting the presence of an active transporter, and the latter indicating passive diffusion representing a minor contribution to the overall acetic acid uptake.

Membranes have proven to be an important target for stress adaptation [48]. Tolerance to various cellular stresses such as D-limonene stress [49], salt stress [50], and hypoxic growth in sugar-rich media lacking lipid nutrients [51] have all been related to changes in membrane lipid composition. The major lipidome rearrangements occurring in *Z. bailii* upon acetic acid exposure suggest that lipid composition is an important factor in acetic acid tolerance. Two genome-wide screens that reported genes required for acetic acid tolerance, presented genes involved in lipid metabolism, including those regulating sphingolipid levels, which further indicates a relationship between lipid composition and acetic acid stress [52], [53].

**Figure 5:**
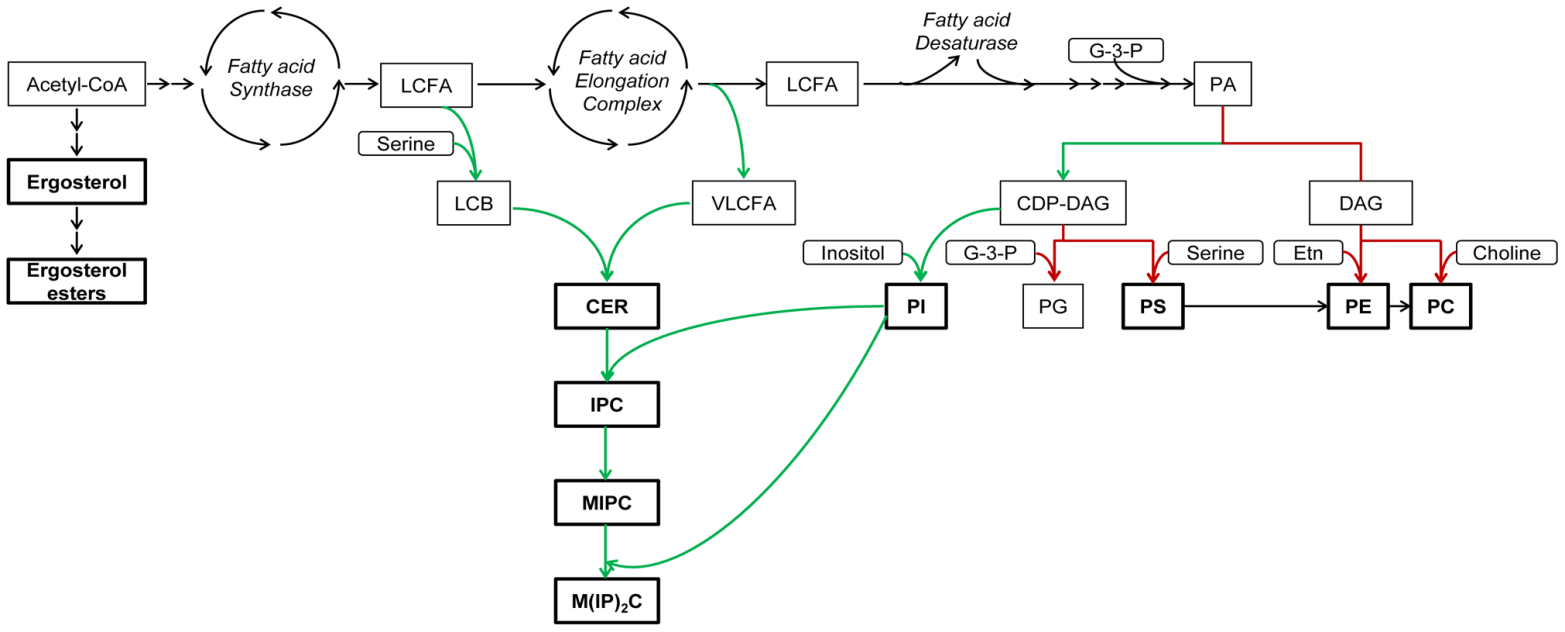
Lipid metabolism in *S. cerevisiae*. Simplified illustration. It is speculated that this illustration is applicable also to *Z. bailii*, due to the high degree of evolutionary conservation of the lipid metabolism. Boxes in bold indicate lipid classes analyzed in this study. Green/red arrows indicate a suggested higher/lower flux in *Z. bailii* resulting from acetic acid stress. Abbreviations: LCFA: long-chain fatty acids, LCB: long-chain base, VLCFA: very-long-chain fatty acids, G-3-P: glycerol-3-phosphate, Etn: ethanolamine. Illustration modified from [35] based on information from the Saccharomyces Genome Database (www.yeastgenome.org). For lipid nomenclature, see Table 1.

Complex sphingolipids were identified in the present study as an important lipid class in the response to acetic acid stress, with considerable increases in both *S. cerevisiae* (IPC 6.2×, MIPC 9.1× and M(IP)_2_C 2.2×) and *Z. bailii* (IPC 4.9×, MIPC 2.7× and M(IP)_2_C 2.7× ), when cultured in the presence of acetic acid. Even more importantly, the basal levels of complex sphingolipids were much higher in *Z. bailii* than in *S. cerevisiae*, further emphasizing the proposed link between high sphingolipid levels and acetic acid tolerance. IPC and MIPC were 14.3× and 22.9× higher in *Z. bailii*, while M(IP)_2_C was 0.9× lower. Since sphingolipids and glycerophospholipids both use fatty acids as precursors, the substantial reduction in PC, PE and PS observed in *Z. bailii* when exposed to acetic acid could possibly be interpreted as a coordinated effort to enrich sphingolipids, using the glycerophospholipids to secure the supply of fatty acids for the synthesis of sphingolipids. However, PI remained unaltered, which is reasonable, as a strategy to increase sphingolipids because their inositol head group is donated from PI. Another consequence of the relative increase in PI is an increase in the level of saturated acyl chains in glycerophospholipids, which also plays a role in changing the plasma membrane properties. A simplified illustration of the lipid metabolism in *S. cerevisiae* is presented in Figure 5, which also indicates changes suggested to take place in *Z. bailii* when cultured with acetic acid.

In contrast, *S. cerevisiae* demonstrated no significant changes in glycerophospholipids, indicating a reduced capacity for membrane composition adaptation compared to *Z. bailii*. The small changes in glycerophospholipids observed are, to a certain extent, consistent with previous studies on the lipidomic profile of *S. cerevisiae* exposed to acetic acid [16], [54]–[56]. However, only data on glycerophospholipids were reported in these four hitherto published studies, and the effect on the other major lipid classes, sphingolipids and sterols in response to acetic acid remains uncharacterized In our study, we found a 0.54× decrease in total sterols in *S. cerevisiae* cultured with acetic acid, compared to control cells. This might appear counter-intuitive, since sterols are generally thought to contribute to bilayer thickness [57], and are often involved in stress resistance, for example, to ethanol [58], osmotic dehydration [59] and D-limonene [49]. Part of the observed decrease in ergosterol may have been compensated by the increase in sphingolipid levels, as it has been suggested that sphingolipids can replace sterols in the membrane [60].

It is generally considered that longer fatty acyl chains and a higher degree of saturation contribute to a more rigid membrane [48]. However, the physical and biological properties of the plasma membrane must be investigated further to ascertain any potential justification of the observed elevated levels of complex sphingolipids in relation to acetic acid stress. Complex sphingolipids, with their saturated long-chain bases (usually 16–18 carbons), and very-long-chain fatty acids (usually 24–26 carbons), have previously been indicated to promote a thicker, and less permeable membrane [61]. In addition, sphingolipids are crucial for the attachment of lipid-anchored proteins to the membrane [62]. Strains lacking sphingolipids are unable to grow at low pH [63], probably due to the fact that the essential Pma1 ATPase proton translocation pump is unable to obtain an active fold and integrate in a membrane lacking sphingolipids [64]. In a study in which *S. cerevisiae* was evolved for acetic acid resistance, Pma1 exhibited a higher *in vivo* activity, although no mutation was detected in the *PMA1* gene [17]. The authors suggested that the higher membrane integrity of the evolved strain could be the explanation. Thus, the potentially positive effect of sphingolipids on acetic acid tolerance may be a combination of lower membrane permeability and enhanced support for membrane proteins. In fact, lipid composition is known to act as an important mediator in determining and fine tuning the activity of membrane proteins [65].

## Conclusions

The lipidome of *Z. bailii* is highly adaptable to acetic acid exposure, while that of *S. cerevisiae* is more stable under the conditions employed in the present study. Saturated glycerophospholipids and sphingolipids appear to be key lipid classes in response to acetic acid stress. *Z. bailii* exhibited a considerable increase in the saturation of glycerophospholipids, as well as an increase in sphingolipids, while *S. cerevisiae* exhibited a large increase only in sphingolipids. Moreover, the higher basal levels of sphingolipids in *Z. bailii* than in *S. cerevisiae* could possibly explain the higher acetic acid tolerance of *Z. bailii*. The present study demonstrates the importance of analyzing glycerophospholipids, sphingolipids and sterols simultaneously to allow conclusions to be drawn from lipidomic changes. Increased sphingolipid synthesis is suggested as a target to increase acetic acid tolerance in *S. cerevisiae*, which would have positive implications on fermentation performance in processes using lignocellulosic hydrolysate.

## Supporting Information

Figure S1 **Phosphatidylcholine species of** ***S. cerevisiae*** **and** ***Z. bailii*** **in response to acetic acid. A.** All results. **B.** Y-axis enlarged at low values. Cells were cultured in minimal medium with and without acetic acid. Apparent quantities were calculated relative to the appropriate internal standard, and normalized to the total amount of phosphate in each sample (see Materials and Methods). The results were calculated from biological replicates (n = 4) and are given as the mean ± standard deviation.
(TIF)

Figure S2 **Phosphatidylethanolamine species of** ***S. cerevisiae*** **and** ***Z. bailii*** **in response to acetic acid. A.** All results. **B.** Y-axis enlarged at low values. Cells were cultured in minimal medium with and without acetic acid. Apparent quantities were calculated relative to the appropriate internal standard, and normalized to the total amount of phosphate in each sample (see Materials and Methods). The results were calculated from biological replicates (n = 4) and are given as the mean ± standard deviation.
(TIF)

Figure S3 **Phosphatidylinositol species of** ***S. cerevisiae*** **and** ***Z. bailii*** **in response to acetic acid. A.** All results. **B.** Y-axis enlarged at low values. Cells were cultured in minimal medium with and without acetic acid. Apparent quantities were calculated relative to the appropriate internal standard, and normalized to the total amount of phosphate in each sample (see Materials and Methods). The results were calculated from biological replicates (n = 4) and are given as the mean ± standard deviation.
(TIF)

Figure S4 **Phosphatidylserine species of** ***S. cerevisiae*** **and** ***Z. bailii*** **in response to acetic acid. A.** All results. **B.** Y-axis enlarged at low values. Cells were cultured in minimal medium with and without acetic acid. Apparent quantities were calculated relative to the appropriate internal standard, and normalized to the total amount of phosphate in each sample (see Materials and Methods). The results were calculated from biological replicates (n = 4) and are given as the mean ± standard deviation.
(TIF)

Figure S5 **Ceramide species of** ***S. cerevisiae*** **and** ***Z. bailii*** **in response to acetic acid. A.** All results. **B.** Y-axis enlarged at low values. Cells were cultured in minimal medium with and without acetic acid. Apparent quantities were calculated relative to the appropriate internal standard, and normalized to the total amount of phosphate in each sample (see Materials and Methods). Abbreviations: DHC: Dihydroceramide, PHC: Phytoceramide. -A,-B, -C, and -D denote one to four hydroxyl groups, respectively. The results were calculated from biological replicates (n = 4) and are given as the mean ± standard deviation.
(TIF)

Figure S6 **Complex sphingolipid species of** ***S. cerevisiae*** **and** ***Z. bailii*** **in response to acetic acid. A.** All results. **B.** Y-axis enlarged at low values. Cells were cultured in minimal medium with and without acetic acid. Apparent quantities were calculated relative to the appropriate internal standard, and normalized to the total amount of phosphate in each sample (see Materials and Methods). -A,-B, -C, and -D denote one to four hydroxyl groups, respectively. The results were calculated from biological replicates (n = 4) and are given as the mean ± standard deviation.
(TIF)

Data S1 **Complete dataset from the lipidome analysis.** The spreadsheet contains raw data from all biological replicates and the statistical parameters mean, median, standard deviation and variance.
(XLSX)
