# Supplementary figures and images for: Lipidomic Profiling of Saccharomyces cerevisiae and Zygosaccharomyces bailii Reveals Critical Changes in Lipid Composition in Response to Acetic Acid Stress
Source: PLoS One. 2013 Sep 4;8(9):e73936. doi: 10.1371/journal.pone.0073936 (PMC3762712; doi:10.1371/journal.pone.0073936)

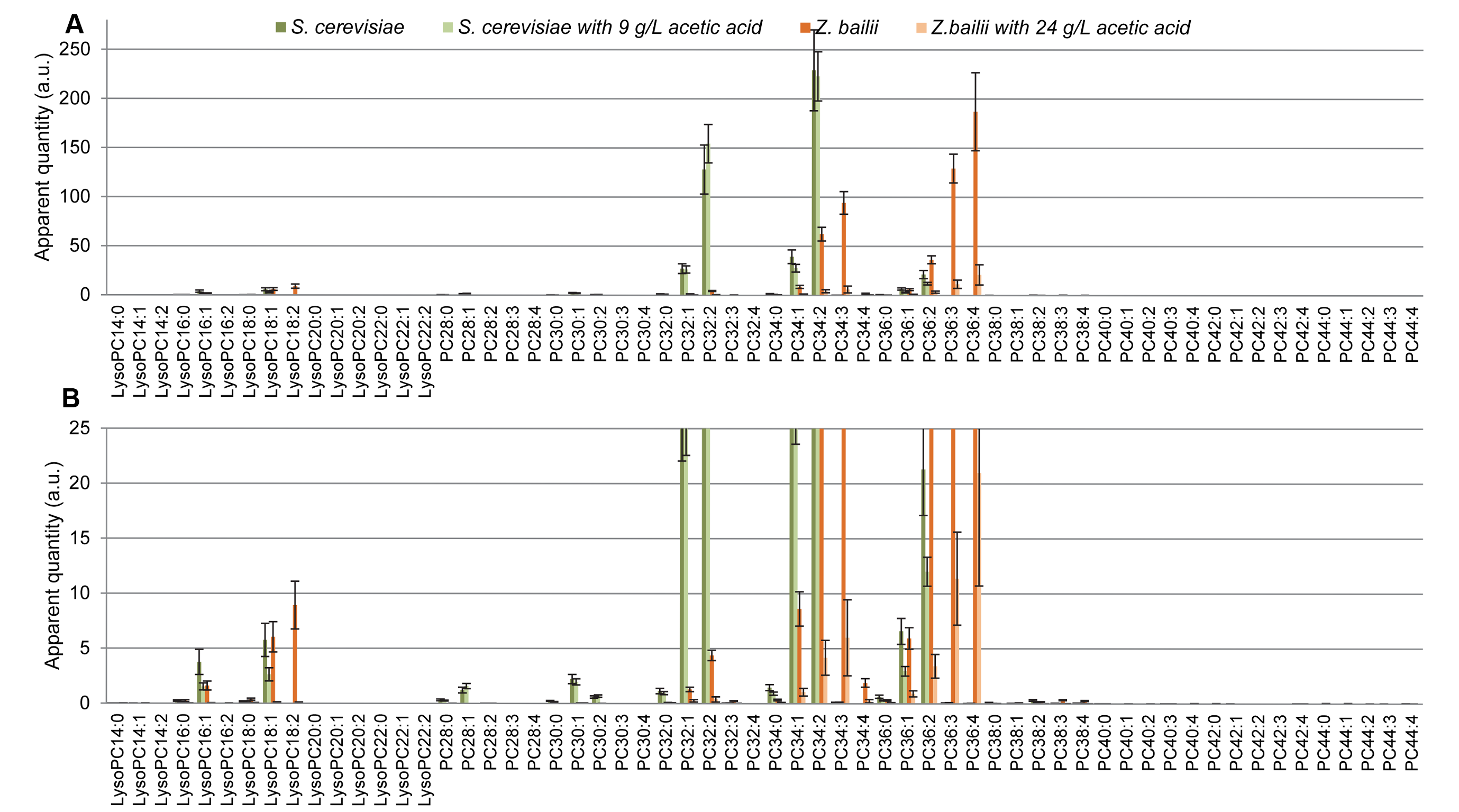

Supplement: Figure S1 — Phosphatidylcholine species of S. cerevisiae and Z. bailii in response to acetic acid. A. All results. B. Y-axis enlarged at low values. Cells were cultured in minimal medium with and without acetic acid. Apparent quantities were calculated relative to the appropriate internal standard, and normalized to the total amount of phosphate in each sample (see Materials and Methods). The results were calculated from biological replicates (n = 4) and are given as the mean ± standard deviation. (TIF) [file pone.0073936.s001.tif]

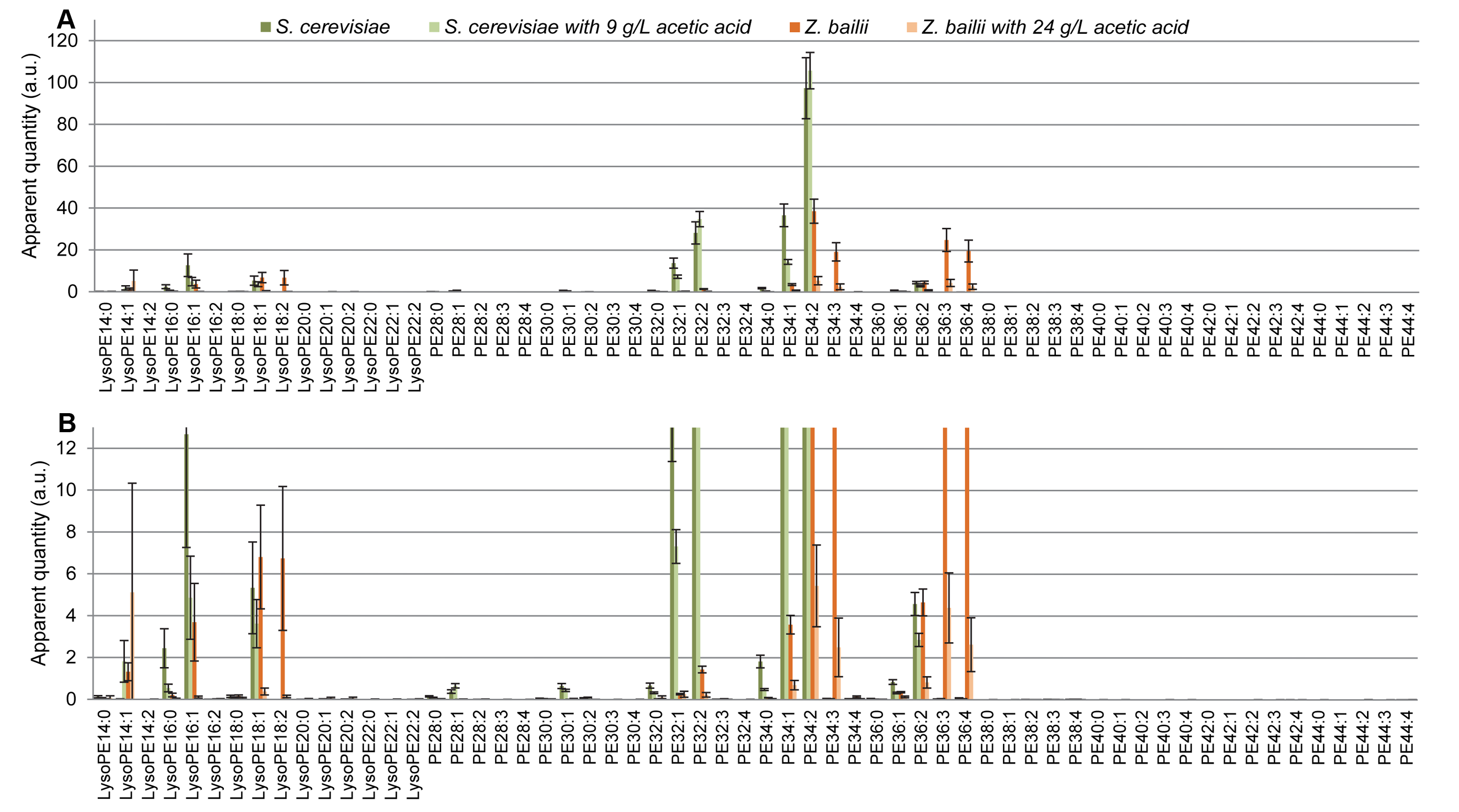

Supplement: Figure S2 — Phosphatidylethanolamine species of S. cerevisiae and Z. bailii in response to acetic acid. A. All results. B. Y-axis enlarged at low values. Cells were cultured in minimal medium with and without acetic acid. Apparent quantities were calculated relative to the appropriate internal standard, and normalized to the total amount of phosphate in each sample (see Materials and Methods). The results were calculated from biological replicates (n = 4) and are given as the mean ± standard deviation. (TIF) [file pone.0073936.s002.tif]

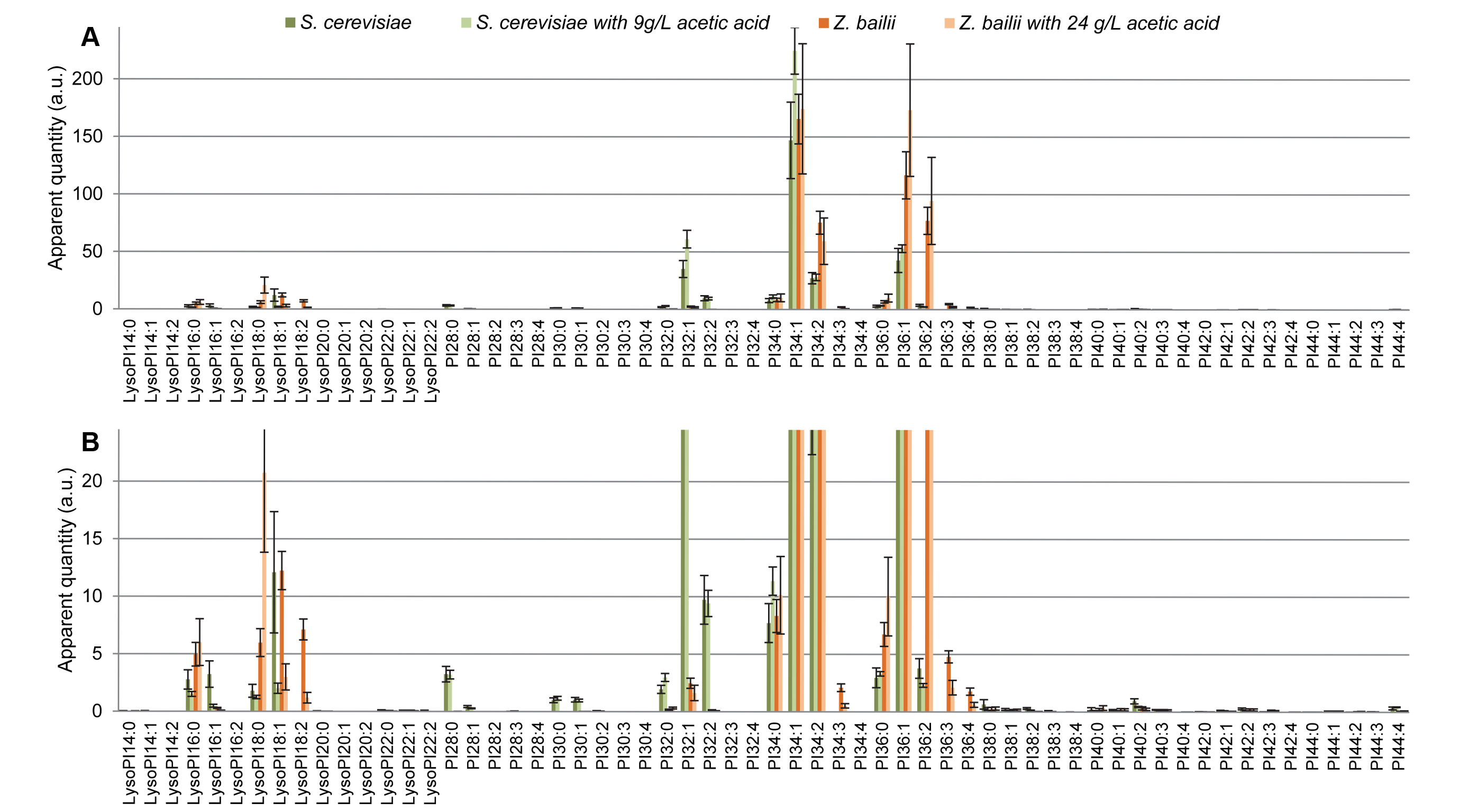

Supplement: Figure S3 — Phosphatidylinositol species of S. cerevisiae and Z. bailii in response to acetic acid. A. All results. B. Y-axis enlarged at low values. Cells were cultured in minimal medium with and without acetic acid. Apparent quantities were calculated relative to the appropriate internal standard, and normalized to the total amount of phosphate in each sample (see Materials and Methods). The results were calculated from biological replicates (n = 4) and are given as the mean ± standard deviation. (TIF) [file pone.0073936.s003.tif]

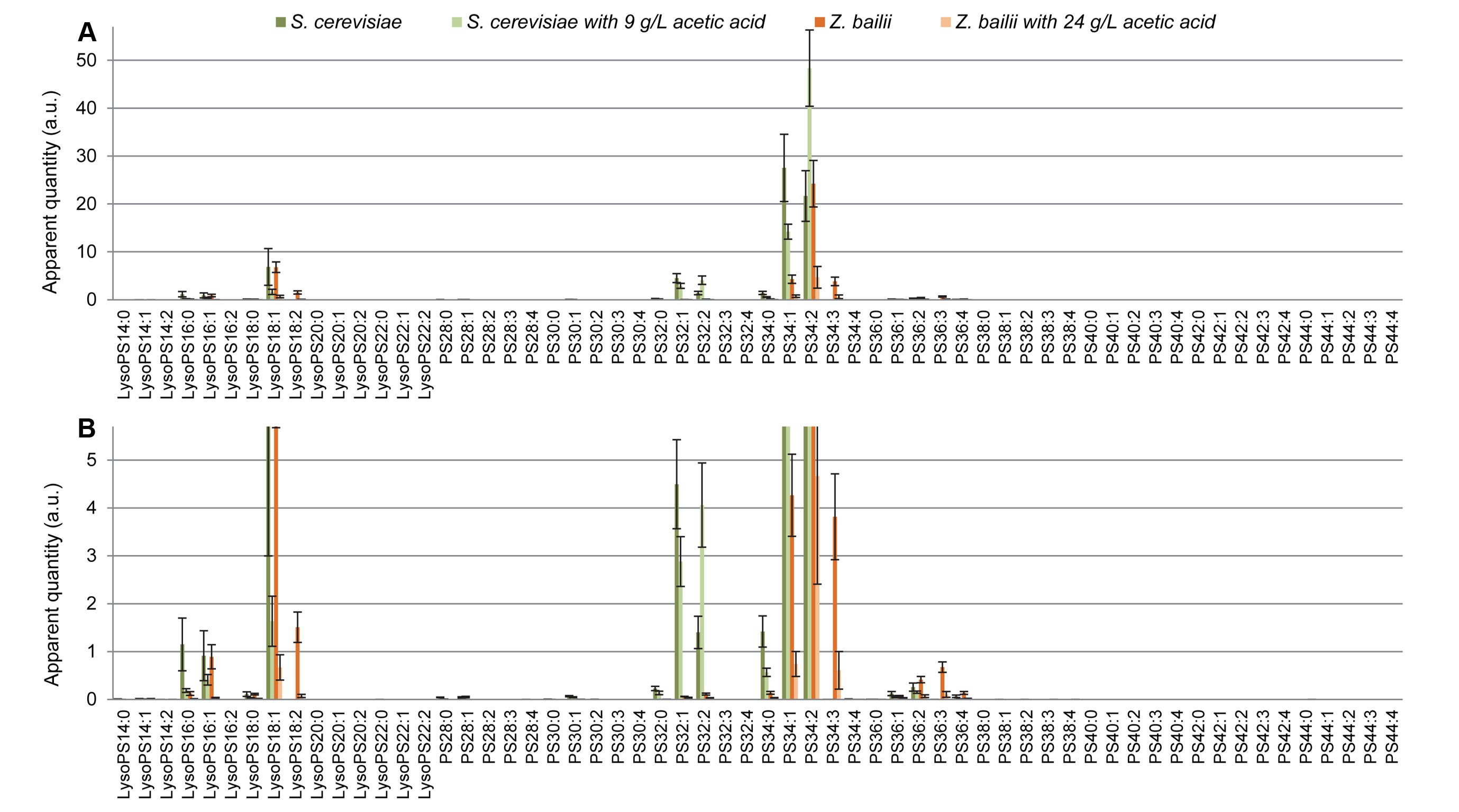

Supplement: Figure S4 — Phosphatidylserine species of S. cerevisiae and Z. bailii in response to acetic acid. A. All results. B. Y-axis enlarged at low values. Cells were cultured in minimal medium with and without acetic acid. Apparent quantities were calculated relative to the appropriate internal standard, and normalized to the total amount of phosphate in each sample (see Materials and Methods). The results were calculated from biological replicates (n = 4) and are given as the mean ± standard deviation. (TIF) [file pone.0073936.s004.tif]

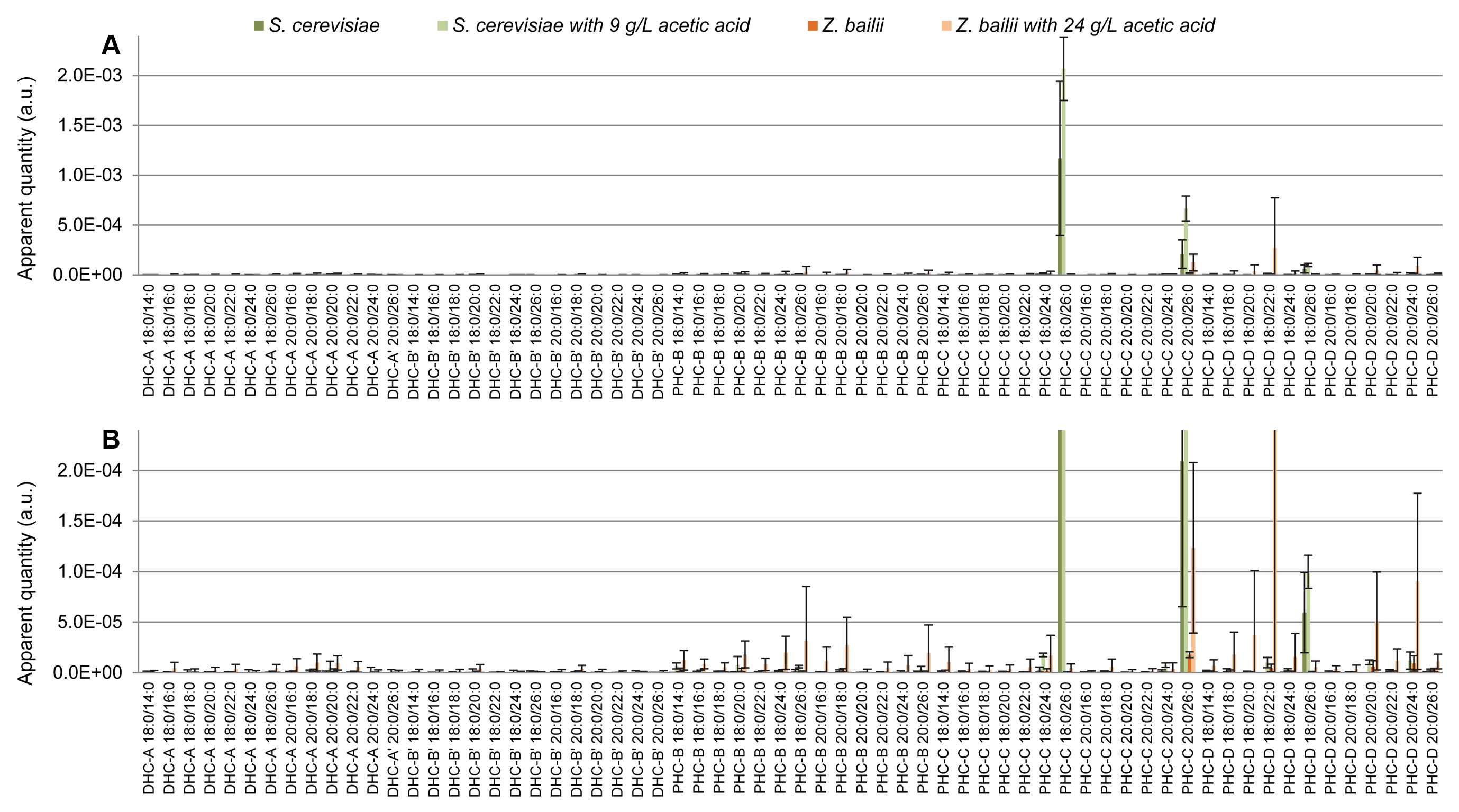

Supplement: Figure S5 — Ceramide species of S. cerevisiae and Z. bailii in response to acetic acid. A. All results. B. Y-axis enlarged at low values. Cells were cultured in minimal medium with and without acetic acid. Apparent quantities were calculated relative to the appropriate internal standard, and normalized to the total amount of phosphate in each sample (see Materials and Methods). Abbreviations: DHC: Dihydroceramide, PHC: Phytoceramide. -A,-B, -C, and -D denote one to four hydroxyl groups, respectively. The results were calculated from biological replicates (n = 4) and are given as the mean ± standard deviation. (TIF) [file pone.0073936.s005.tif]

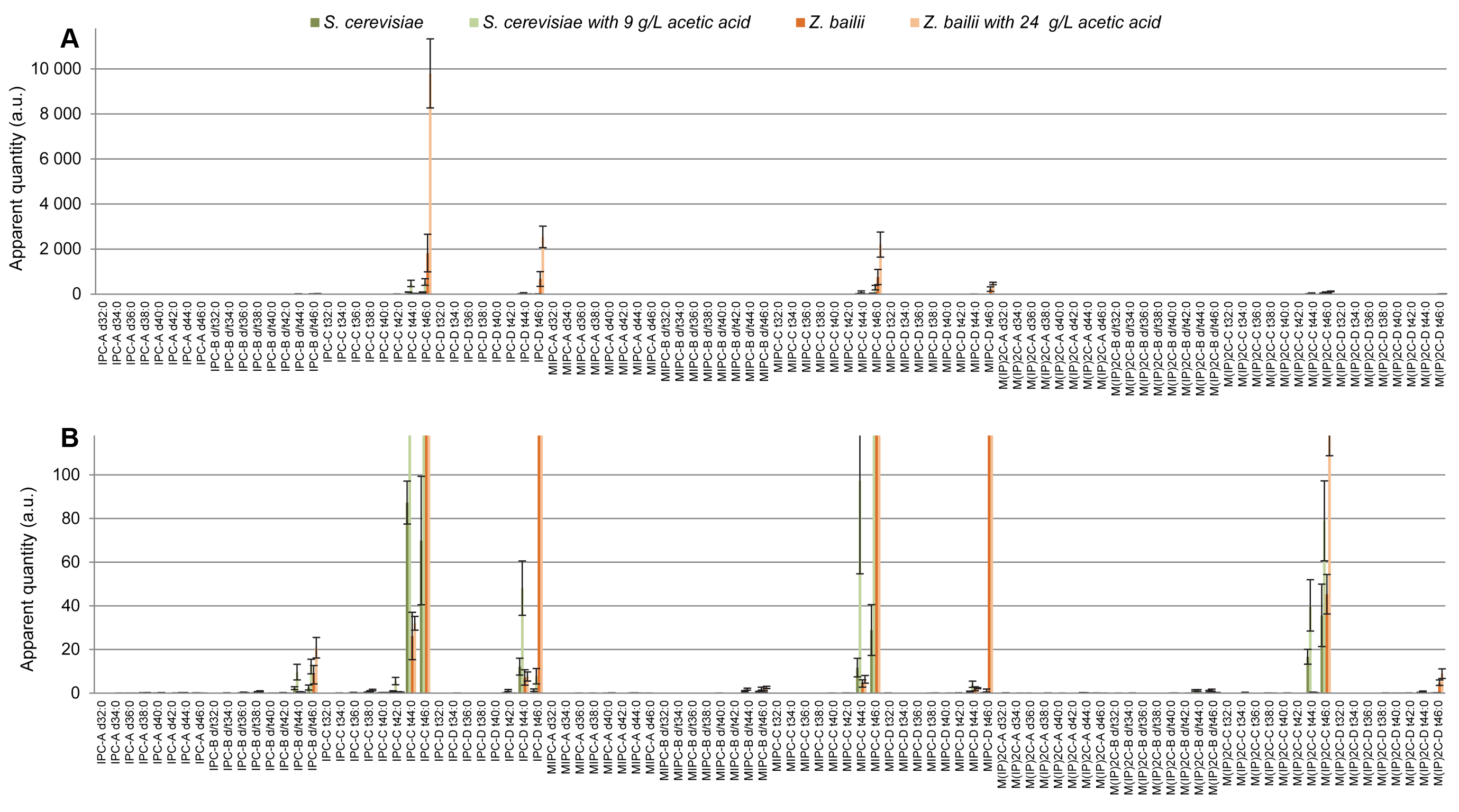

Supplement: Figure S6 — Complex sphingolipid species of S. cerevisiae and Z. bailii in response to acetic acid. A. All results. B. Y-axis enlarged at low values. Cells were cultured in minimal medium with and without acetic acid. Apparent quantities were calculated relative to the appropriate internal standard, and normalized to the total amount of phosphate in each sample (see Materials and Methods). -A,-B, -C, and -D denote one to four hydroxyl groups, respectively. The results were calculated from biological replicates (n = 4) and are given as the mean ± standard deviation. (TIF) [file pone.0073936.s006.tif]
